# Determinants of health disparities between Italian regions

**DOI:** 10.1186/1471-2458-10-296

**Published:** 2010-06-01

**Authors:** Luisa Franzini, Margherita Giannoni

**Affiliations:** 1 Management, Policy, and Community Health Division, University of Texas School of Public Health, 1200 Pressler Drive, Houston, TX 77030, USA; 2 Dipartimento di Economia Finanza e Statistica Universita' degli Studi di Perugia, Via Pascoli 20, 06124, Perugia, Italy; 3 MECOP Institute, University of Lugano,Via Giuseppe Buffi 6, CH-6904 Lugano, Switzerland

## Abstract

**Background:**

Among European countries, Italy is one of the countries where regional health disparities contribute substantially to socioeconomic health disparities. In this paper, we report on regional differences in self-reported poor health and explore possible determinants at the individual and regional levels in Italy.

**Methods:**

We use data from the "Indagine Multiscopo sulle Famiglie", a survey of aspects of everyday life in the Italian population, to estimate multilevel logistic regressions that model poor self-reported health as a function of individual and regional socioeconomic factors. Next we use the causal step approach to test if living conditions, healthcare characteristics, social isolation, and health behaviors at the regional level mediate the relationship between regional socioeconomic factors and self-rated health.

**Results:**

We find that residents living in regions with more poverty, more unemployment, and more income inequality are more likely to report poor health and that poor living conditions and private share of healthcare expenditures at the regional level mediate socioeconomic disparities in self-rated health among Italian regions.

**Conclusion:**

The implications are that regional contexts matter and that regional policies in Italy have the potential to reduce health disparities by implementing interventions aimed at improving living conditions and access to quality healthcare.

## Background

Reports on equity in health in Italy have shown that differences in health are due to social differences, with the lowest social classes characterized by higher perinatal mortality rate, lower self assessed health status, higher chronic illness rates, higher cancer rates and higher mortality rates [1-5]. However, despite having relatively poor average self-reported health, Italy it is one of the Southern European countries with relatively low, but increasing, socioeconomic health inequalities compared to other European countries. In fact, socioeconomic inequalities in health have been increasing faster in Italy compared to other European countries [6,7]. Moreover, out of 13 European countries, Italy was one of the countries where regional income disparities are most pronounced resulting in high regional health disparities [8]. Self-rated health varies greatly by regions with the percentage of residents reporting poor health ranging from 4% in Trentino Alto Adige to 10% in Calabria and Sicilia.

A study using data from the 2000 Italian National Health Interview Survey, looked at geographic variation in subjective health and presence of chronic conditions, focusing on the effects of individual and area-based socioeconomic conditions and their heterogeneity across regions [9]. The study finds a North-South gradient in self-assessed health, affected mainly by area composition with respect to individual education, and only slightly influenced by contextual factors, such as area level socioeconomic and power resources. Another study, based on a relatively small sample of individual households income and health data from Bank of Italy collected in 2004, has found that, although at national level individual income affects positively self-assessed health, there seem not to be a clear socioeconomic gradient in terms of North-South divide [10]. This study, however, did not consider simultaneously the role of regional and individual level characteristics.

As the evidence is not clear, it seems important to further investigate health inequalities in Italian regions. In this paper, we use data from the "Indagine Multiscopo sulle Famiglie", a survey of aspects of everyday life in the Italian population, to investigate the determinants of regional differences in self-reported poor health, after controlling for individual level factors, including individual socioeconomic status [11,12].

### Conceptual framework

The conceptual framework for our study draws on socio-ecological models that postulate that health is influenced by a wide range of factors at multiple levels [13-15]. Determinants of health include socioeconomic factors, social and physical environments, healthcare, and health behaviors [16-18]. Most models identify socioeconomic conditions at the individual level as well as at the group level as the fundamental causes of disease [19,20]. Socioeconomic factors contribute to unequal social and physical environmental exposures which contribute to health inequalities. A large literature discusses the mechanisms that underline the relationship between socioeconomic factors and health at the regional level [21-24]. Two major theories have been proposed: one focuses on material deprivation and the other on social/psychological wellbeing. In the material deprivation interpretation, the negative effects of socioeconomic disadvantage on health operate through a lack of physical resources and underinvestment in infrastructure and services, including housing, environmental quality, and healthcare services [17,21,25]. The second theory emphasizes the role of social/psychological factors, including social capital and social isolation, in the relationship between socioeconomic factors and health [24,26-28].

**Figure 1:**
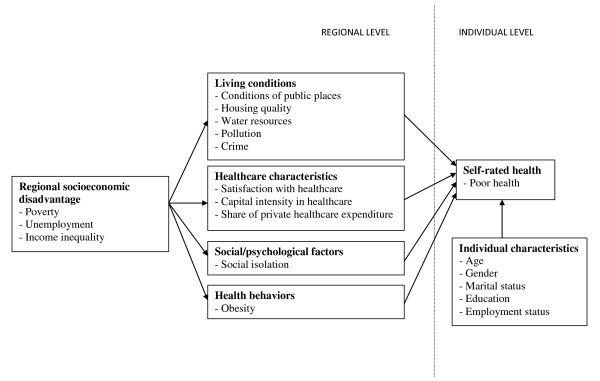
**Conceptual model for the determinants of self-rated health**.

In this paper we consider a socio-ecological model (see Figure 1) where regional socioeconomic factors affect health outcomes through material deprivation, social/psychological factors, and health behaviors measured at the regional level in order to investigate regional level determinants of self-rated health among Italians. All our models control for individual level factors. Our hypothesis is that, in Italy, regional health inequalities reflect regional socioeconomic disadvantage. Furthermore, we hypothesize that regional level socioeconomic disadvantage negatively affect health by impacting the social and physical environments at the regional level. We hypothesize that regions that are more disadvantaged socioeconomically will have: 1) more material deprivation resulting in poorer living conditions and worse healthcare; 2) more social/psychological disadvantage reflected in more social isolation; and 3) more unhealthy behaviors. We will test the hypothesis that regional level poor living conditions, healthcare factors, social isolation, and unhealthy behaviors mediate the impact of regional socioeconomic disadvantage on self-rated health in Italian regions.

## Methods

### Data

The data used for the analysis were collected both at the individual level and at the regional level. At the individual level, data were taken from Multiscopo, a survey on health and living conditions conducted by Istat, the Italian National Institute of Statistics, in the period 2004-2005 [11,12]. Multiscopo interviewed a nationally representative sample of families and individuals in order to describe health and healthcare utilization of the Italian population. Starting in March 2004 until March 2005, 50,474 sampled families (defined as a group of people living together for several possible reasons) and 128,040 individuals, distributed in 1,465 Italian municipalities, were interviewed. The survey non response rate was 14%. Missing values were imputed using donors imputation method. The overall survey imputation rate for missing values was 2.5% (but only 1.8% for our dependent variable self assessed health). The Multiscopo sample is representative of the Italian population, with a margin error of 2.9% at national level. Part of the questionnaire was completed by direct interviews and, when individuals were not available, information was gathered from another family member. Part of the questionnaire was self administered. The survey design and methodology is further described by Istat [29]. Regional level variables were taken from Istat data available at the regional level for 2004 and 2005 [11,12,30,31].

Ethics committee approval for this study was not secured given it uses publicly available data made available to researchers by Istat, who produces and disseminates information collected in full compliance with the regulations pertaining to the privacy of respondents. No competing interests were declared.

## Measures

### Individual level measures

Individual level variables were obtained from Multiscopo [11,12]. The dependent variable used is a measure of self-rated health, which is well known to be highly predictive for mortality and the onset of disability [32-34]. Individuals were asked "How is your health in general?" with possible response: very good, good, fairly good, bad, and very bad. Following other researchers [6,35-38], the responses were dichotomized into a categorical variable (poor health) taking the value one for those reporting poor health (bad or very bad) and the value of zero for those not reporting poor health (very good, good, fair).

Individual sociodemographic characteristics included age (17-35, 35-44, 45-64, 65-74 and over 75), gender, marital status (married, separated or divorced, widowed or single), education (university degree or other post-graduate qualifications, high school or secondary school diploma, less than high school), and employment status (employed, self-employed, including professionals, retired, other not working, including the unemployed and those not in the labor force). Given our focus on regional factors, individual level variables can be considered as confounding variables in our models.

### Regional level measures

Regional characteristics, obtained from Istat, include socioeconomic factors (socioeconomic disadvantage), material deprivation (living conditions and healthcare), social/psychological factors (social isolation), and health behaviors (obesity) [11,12,30,31].

Regional level socioeconomic disadvantage was measured by a scale using three indicators: poverty, unemployment, and income inequality, measured by the Gini coefficient [31]. Poverty was defined as the percentage of individuals in the region whose monthly consumption expenditures are below the relative poverty line, which is defined by Istat using household expenditure survey data (e.g., for 2008 this was set to 999.67 euro for a two members family) [30]. The Gini was computed by Istat [39]. These variables were standardized and averaged to create a measure of socioeconomic disadvantage with zero mean and standard deviation equal to one. The socioeconomic disadvantage scale had good internal reliability with a Cronbach's alpha of 0.90 [40].

Regional level material deprivation was assessed by a scale reflecting living conditions and by characteristics of the healthcare sector. Living conditions were measured by the proportion of families reporting that were either a fair number or many problems in the area in which they lived, in terms of conditions of public places, housing quality, water resources, pollution, and crime [12]. The poor living conditions scale (alpha = 0.85) consisted of 12 items representing the perceived conditions of daily living: conditions of public places (dirty streets, parking difficulty, traffic, no public lights in streets, streets in poor condition), housing quality (small residential unit, residential unit far from family, residential unit in poor condition), water availability and quality (irregular water service, does not drink tap water), pollution (air pollution) and crime.

Characteristics of the healthcare sector at the regional level were measured by a scale for healthcare satisfaction, a healthcare capital intensity index, and the share of private healthcare expenditure. Satisfaction with healthcare was measured by reported satisfaction with their hospital stay for residents who had at least one hospital stay in the last three months (3.2% of sample). The healthcare satisfaction scale (alpha = 0.90) had 4 items: percentage of respondents who were highly satisfied with physicians, with nurses, with room and board, and with hygiene [12]. Capital intensity in the healthcare sector was captured by an index that represents the number of medical equipment machines per resident in the region. The number of 18 types of medical machines, such as MRIs, dialysis equipment, ventilators, radiology equipment, and anesthesia equipment, is reported by Istat for each region [30]. Share of private healthcare expenditure was measured as the percentage of total healthcare expenditure that was private expenditure [30].

Social/psychological disadvantage was assessed by social isolation measured as the percentage of residents in a region who reported not having any friends [12]. Finally, we used regional obesity rates as a proxy for health behaviors since obesity often results from unhealthy behaviors such as poor diet and lack of physical activity [30].

### Statistical analysis

Descriptive statistics were computed for individual and regional variables. Multilevel models were used to model the relationship between reporting poor health (bad/very bad health) and individual and regional characteristics. Multilevel methods, developed for use with nested data structures, are found in many areas of research that investigate contextual level effects [41,42]. We used two-level multilevel models where the individual level was level 1 and regional level was level 2. We did not introduce the household level in the models because the majority of respondents (72%) lived in households with 2 or fewer respondents. Given that the outcome variable (reporting poor health) is categorical, we estimated a multilevel logistic regression with Stata software [43]. We report odds ratios with their 95% confidence interval. The odds ratios for categorical variables (coded zero and one) represent the odds of being in poor health for the exposure (one) category divided by the odds of being in poor health for the referent (zero) category. For continuous variables, the odds ratios represent the odds of reporting poor health for a one-unit increase in the continuous variable. In our analysis, we rescaled regional level scales, the share of private healthcare expenditures, and obesity rates so that the odds ratios represent the odds of reporting poor health for a 10-unit increase. Regional level variance and the inter-correlation coefficient (ICC), representing the percentage of total variance in poor health attributable to regional level variance, are also reported.

Mediation was tested using the causal step approach which specifies a series of tests in a causal chain. The causal step approach to test if Z mediated the effect of X on Y consists of (1) regressing X on Y and (2) regressing Z and X on Y. If two conditions are met (the coefficient of X on Y in (1) is significant, the coefficient of Z on Y in (2) is significant while the coefficient of X on Y in (2) is not significant), Z is said to mediate the effect of X on Y [44,45]. A more stringent test, proposed by Judd and Kenny, adds a third regression, (3) regress Z on X, and requires the coefficient of Z on X in (3) to be significant [46,47]. Such mediation tests, when used with observational data, demonstrate that the causal processes hypothesized in the model are consistent with the data but do not prove causality.

## Results

### Sample description

Table 1 describes the individual level sociodemographic characteristics of the sample. The 7% prevalence of poor health in this Italian sample is consistent with Carrieri [10] and van Doorslaer & Jones [48] who used Bank of Italy data (2004) and the European Community Household Panel (1996) respectively, but were lower than the prevalence reported in the Italian Health Interview Survey [6,38]. The characteristics of the 20 Italian regions are described in Table 2. Most regional measures vary varied greatly between regions, in particular, socioeconomic measures such as poverty rates that range from 4% to 27% and unemployment rates that range from 2% to 23%.

**Table 1:** Description of individual level characteristics (N = 107,087)

Individual characteristics	Percentage (%)	Frequency (N)
*Age group*		
17-34	26%	28,351
35-44	19%	20,140
45-64	31%	33,438
65-74	13%	13,656
75 and over	11%	11,502

*Gender*		
Male	48%	51,072
Female	52%	56,015

*Marital status*		
Married	57%	61,367
Separated/divorced	5%	5,685
Widowed	10%	10,321
Single	28%	29,714

*Education*		
University degree or higher	9%	9,743
High school	31%	33,724
Less than high school	59%	63,620

*Employment status*		
Employee	33%	35,413
Self-employed	12%	12,517
Retired	20%	21,206
Not working/other	35%	37,951

*Self-rated health*		
Very good	17%	18,228
Good	42%	45,202
Neither good nor bad	34%	35,989
Bad	6%	6,281
Very bad	1%	1,387
*Poor health (bad/very bad)*	*7%*	*7,668*

**Table 2:** Description of regional level characteristics for the 20 Italian regions

Regional Characteristics	Mean	Standard deviation	Min	Max	Cronbach's Alpha
***Economic disadvantage^1^***	*0.00*	*0.91*	*-1.01*	*1.71*	*0.90*
Poverty rate	0.13	0.08	0.04	0.27	
Unemployment rate	0.09	0.07	0.02	0.23	
Gini coefficient	0.29	0.03	0.25	0.33	

***Material deprivation***					
*Poor living conditions^1^*	*0.29*	*0.05*	*0.17*	*0.39*	*0.85*
Dirty streets*^2^*	0.30	0.09	0.15	0.49	
Difficulty parking*^2^*	0.38	0.09	0.28	0.57	
Traffic*^2^*	0.42	0.10	0.25	0.60	
No public lights in streets*^2^*	0.30	0.06	0.19	0.39	
Streets in poor conditions*^2^*	0.43	0.09	0.23	0.57	
Small residential unit*^2^*	0.12	0.03	0.10	0.18	
Residential unit far from family*^2^*	0.21	0.05	0.11	0.32	
Residential unit in poor conditions*^2^*	0.05	0.02	0.03	0.10	
Irregular water service*^2^*	0.14	0.09	0.02	0.36	
Does not drink tap water*^2^*	0.34	0.14	0.05	0.65	
Air pollution*^2^*	0.34	0.12	0.13	0.57	
Crime*^2^*	0.24	0.11	0.12	0.53	
*Satisfaction with healthcare^1^*	*0.29*	*0.10*	*0.15*	*0.50*	*0.90*
Highly satisfied with physicians*^2^*	0.35	0.13	0.14	0.56	
Highly satisfied with nurses*^2^*	0.34	0.13	0.15	0.55	
Highly satisfied with room and board*^2^*	0.22	0.10	0.12	0.43	
Highly satisfied with hygiene*^2^*	*0*.29	*0*.12	*0*.11	*0*.49	
*Capital intensity in healthcare^3^*	*23.35*	*4.92*	*15.05*	*34.14*	
*Share of private healthcare expenditure*	*0.38*	*0.21*	*0.04*	*0.77*	

***Social/psychological factors***					
*Social isolation (no friends^2^)*	*0.05*	*0.02*	*0.03*	*0.08*	

***Health behaviors***					
*Obesity rate*	*0.10*	*0.02*	*0.07*	*0.13*	

### Multilevel models

Estimates for the multilevel logit regressions are reported in Table 3. Model 1 includes only individual level factors (age, gender, marital status, education, employment status). Individual determinants of self-rated health were consistent with those in the other studies of self-rated health in Italy and elsewhere: health decreased with age and increased with education; non married individuals reported worse health than married individuals; and those who were working, either as employee or self-employed, reported better health than those who were not working [8,35,36,38,49].

**Table 3:** Odds ratios and 95% confidence intervals from multilevel logistic regressions of poor health on individual and regional characteristics N = 107,087

Dependent variable: Poor health	Model 1		Model 2		Model 3	
	OR	95% CI	OR	95% CI	OR	95% CI
***Individual level factors***						
*Age*						
Less than 35	0.12	(0.10, 0.14)	0.12	(0.10, 0.14)	0.12	(0.10, 0.14)
35-44	0.37	(0.33, 0.42)	0.37	(0.33, 0.42)	0.37	(0.33, 0.42)
45-64	ref		ref		ref	
65-74	1.82	(1.69, 1.96)	1.82	(1.69, 1.96)	1.82	(1.69, 1.96)
75 and over	3.74	(3.47, 4.03)	3.74	(3.47, 4.03)	3.74	(3.47, 4.03)
*Gender*						
Female	ref		ref		ref	
Male	1.00	(0.94, 1.07)	1.00	(0.94, 1.07)	1.00	(0.94, 1.07)
*Marital status*						
Married	ref		ref		ref	
Separated/divorced	1.43	(1.26, 1.61)	1.43	(1.26, 1.62)	1.43	(1.26, 1.62)
Widowed	1.28	(1.20, 1.37)	1.28	(1.20, 1.37)	1.28	(1.20, 1.37)
Single	1.41	(1.29, 1.53)	1.41	(1.29, 1.53)	1.41	(1.29, 1.53)
*Education*						
College degree	ref		ref		ref	
High school	1.16	(0.99, 1.36)	1.16	(0.99, 1.36)	1.16	(0.99, 1.36)
Less than high school	2.07	(1.79, 2.39)	2.07	(1.79, 2.39)	2.07	(1.79, 2.39)
*Employment status*						
Employee	ref		ref		ref	
Self-employed	0.73	(0.62, 0.86)	0.73	(0.62, 0.86)	0.73	(0.62, 0.86)
Retired	2.17	(1.95, 2.41)	2.17	(1.95, 2.41)	2.17	(1.95, 2.42)
Not working/other	3.01	(2.72, 3.34)	3.01	(2.71, 3.33)	3.00	(2.71, 3.33)
***Regional level factors***						
Economic disadvantage^1^			1.21	(1.09,1.34)	0.99	(0.84, 1.12)
Poor living conditions^1^					1.41	(1.04, 1.92)
Satisfaction with healthcare^1,3^					0.96	(0.85, 1.08)
Capital intensity in healthcare^2,3^					1.00	(0.97, 1.02)
Share of private healthcare expenditure^1^					0.94	(0.88, 0.99)
Social isolation (no friends)					0.99	(0.91, 1.09)
Obesity rate^1^					1.24	(0.61, 2.54)
						
Region level variance	0.07	(0.03, 0.13)	0.04	(0.02, 0.08)	0.03	(0.01, 0.05)
ICC	0.020	(0.010, 0.038)	0.012	(0.006, 0.024)	0.008	(0.004, 0.016)

^1^: Rescaled so that OR represent change in poor health associated with a 10% change in regional factor.

^2^: Number of medical equipment machines per 10,000 residents

^3^: These variables are measured so that larger values represent a beneficial effect on health (and a negative effect on poor health).

Model 2 adds the index for socioeconomic disadvantage at the regional level. Living in a region with a standard deviation higher socioeconomic disadvantage increased the odds of reporting poor health by 21%. Similar results are obtained by estimating the model with each indicator of regional socioeconomic disadvantage separately. A 10% increase in poverty rate increased the odds of reporting poor health by 19% (OR 1.19, 95% CI: 1.05, 1.35), a 10% increase in unemployment rate increased the odds of reporting poor health by 32% (OR 1.32, 95% CI: 1.17, 1.49), and a 0.10 increase in income inequality as measured by the Gini coefficient increased the odds of reporting poor health by 70% (OR 1.70, 95% CI: 1.14, 2.53).

Model 3 adds other regional factors (living conditions, satisfaction with healthcare, capital intensity in healthcare, share of private health expenditures, social isolation, and obesity rate) which may mediate the effects of socioeconomic status on poor health. The odds ratio of economic disadvantage decreases from 1.21 to 0.99 and is no longer statistically significant, but living in regions with worse living conditions (by 10%) increased the odds of reporting poor health by 41% and living in regions with a larger share of private healthcare expenditures (by 10%) decreased the odds by 6%. These results indicate that poor living conditions and share of private healthcare expenditures mediate the association of regional socioeconomic status to poor health. The OR for the individual level factors are unchanged in models 2 and 3 after adding the regional level factors. We also looked at the role of individual regional variables by running the regressions where each regional variable was entered separately (not reported). While each regional variable reduced the coefficient of economic disadvantage somewhat, only satisfaction with healthcare reduced it to the point of non significance.

Using the more stringent Judd and Kenny [46] test, we estimated the regression of socioeconomic disadvantage on living conditions, satisfaction with healthcare, capital intensity in healthcare, share of private healthcare expenditures, social isolation, and obesity rates in the 20 regions (Table 4). While all the coefficients were the expected sign, only share of private healthcare expenditures reached statistical significance. Therefore the Judd and Kenny [46] test supports the hypothesis that regional share of private healthcare expenditures mediates the effects of socioeconomic disadvantage on poor health.

**Table 4:** Mediation regression: OLS regression of economic disadvantage (20 regions)

Dependent variable: Economic disadvantage	Coefficient	CI 95%
		
Poor living conditions	0.46	(-0.12, 1.03)
Satisfaction with healthcare	-0.10	(-0.33, 1.30)
Capital intensity in healthcare^1^	-0.21	(-0.73, 0.30)
Share of private healthcare expenditure	-0.12	(-0.23, -0.01)
Social isolation	-0.22	(-2.02, 1.58)
Obesity rate	1.13	(-0.22, 2.47)
		
*Adjusted R^2^= 0.64*		

^1^: Number of medical equipment machines per 10,000 residents

We obtained similar results when analyzing each indicator of regional socioeconomic disadvantage separately. In models 2 and 3 with poverty and Gini entered separately, living conditions and share of private healthcare expenditures mediate the association of regional socioeconomic status to poor health, but we found no mediation in the models with unemployment.

In the empty model which includes only the constant term (not reported), the ICC for poor health was statistically significant at 1.8%, implying that 1.8% of the total variation in poor health was due to variation in poor health between regions. After adjusting the ICC for individual level factors (model 1), the ICC was still statistically significant at 2.0, implying that differences in poor health by region were not due to compositional effects. The ICC decreased after adding regional factors (model 2 and 3), but remained statistically significant, indicating that the regional factors included in our models did not fully account for regional variations in poor health.

## Discussion

We found significant disparities in self-rated health by regional socioeconomic status in Italy. Residents living in regions with more poverty, more unemployment, and more income inequality were more likely to report poor health. This is consistent with studies in other countries, in particular the United States [27,35]. However, regional low socioeconomic status ceased to be significant when regional living conditions, healthcare, social isolation, and health behaviors were added to the model. In particular, variables reflecting living conditions and healthcare factors were significant and mediated socioeconomic disparities in health status among Italian regions. A more stringent test of mediation, the Judd and Kenny [46] test, supported that private share of healthcare expenditures mediated socioeconomic differences in self-rated health. The lack of significance of living conditions in the more stringent test could be due to the small sample size (20 regions).

Poor living conditions, which have the highest impact with an OR of 1.41, are likely to affect self-rated heath through several mechanisms. The stress of daily life is increased by hassles such as difficulty parking, traffic, living away from family, and poor public services (e.g. irregular water and dirty and unlit streets). Higher crime, actual or perceived, make residents feel unsafe and increases stress. Higher stress often leads to worse health [50,51]. Poor quality housing and poor conditions of public places can impact both physical health as well as mental wellbeing. For example, individuals living in small, overcrowded, and damp homes are more likely to get sick. So are those living on dirty streets, where trash collection may be infrequent. Pollution and poor water quality also have the potential for impacting physical health directly. Improving daily living conditions was identified as an overreaching principle to reduce health inequalities by The Commission on Social Determinants of Health set up by the WHO [18].

The proportion of private expenditure of total healthcare expenditure affects positively self-rated health, though the effect estimate is lower than for poor living conditions. The Italian health care system is characterized by regional decentralization coupled with a welfare-mix model, with the public sector becoming weaker over time in its capacity to provide high quality health care and outsourcing services to both private and non-profit sector. Despite the existence of a universal health care system, private expenditure in 2008 accounted for 23% out of total medical expenditure in Italy, of which 85% is out-of-pocket expenditure used to top-up publicly provided services and gain access to faster and better quality private care [52]. Higher per capita total health care expenditure characterize the richer Northern Regions, whereas the majority of Southern and Central regions show levels of both public and private expenditure below the national average [53]. As access to quality healthcare ultimately affects health status of individuals, our finding of a positive effect of private expenditure on health, after adjusting for structural and socio-economic differences across regions, could reflect problems of equity in access to private and often better quality and faster access healthcare in Italy.

Remaining regional differences in health, after taking the socioeconomic, material, and psychosocial factors into account, could possibly be reduced by using more complete and better measures of regional characteristics (for example cultural differences).

The findings in this study may appear to lend some limited support to the material deprivation interpretation [17,21,25]. The factors found to explain socioeconomic differences in health in this study are mainly 'material' factors which include a lack of physical resources (housing quality, air pollution) and underinvestment in infrastructure (difficulty parking, traffic, unlit streets) and services (healthcare, irregular water service, dirty streets). However, these 'material' factors were measured subjectively, as perceived by the participants, and not objectively. It is therefore rather difficult to disentangle the 'material' effect of, say, poor living conditions, from their 'psychosocial' effect. Also, while social isolation was not significant in our model, it should not be interpreted as evidence against the social/psychological interpretation, as we had very limited data on social/psychological factors. Only one item (having no friends) was used to measure social isolation, but there were no measures for social capital, trust, or measures of socially hazardous environments that have been shown to influence health [23,24].

It is noteworthy that self-rated health variation at the regional level accounted for about 2% of total variation in self-rated health, which is in a range consistent with findings in other studies. For example, variation in health status is 2% at the municipality level in Sweden [54] and is 4% in U.S.A [35]. Our findings imply that regional context matters in explaining regional disparities in health among Italian regions, though the majority of variation is at the individual level. It is also interesting that adding regional factors does not change the influence of individual level characteristics. It indicates that individual and regional factors are independently influencing self-rated health and that individual factors are explaining one part of the variance, and the regional factors explain a separate, non-overlapping part of the variance. Of course, attribution of effects to the individual and regional levels are somewhat approximate because many individual level factors are affected by regional context, but this indirect effect of regional context on health outcomes through individual factors is not well captured in our models.

A limitation of the data is the absence of information on regional level social capital and on individual level income. However, we used education as a proxy for individual level socioeconomic status, which has the advantage of being more stable. Moreover, available evidence based on individual households income and health data, but not considering the role of regional characteristics, confirms our findings using regional aggregate measures [10]. A further limitation is the cross-sectional nature of the data that prevents any causal inference from these data.

## Conclusions

Overall, we found that poor living conditions and private share of healthcare expenditures at the regional level are determinants of socioeconomic disparities in self-rated health among Italian regions. The implications are that regional contexts matter and that regional policies have the potential to reduce health disparities. The results in this study suggest that regions can positively impact health disparities by implementing policies and interventions aimed at improving living conditions and access to quality healthcare.

## Competing interests

The authors declare that they have no competing interests.

## Authors' contributions

MG obtained the data. LF and MG both conceived of the study and performed the statistical analysis. LF drafted the manuscript. Both authors revised the manuscript critically and approved the final manuscript.

## Pre-publication history

The pre-publication history for this paper can be accessed here:

http://www.biomedcentral.com/1471-2458/10/296/prepub
